# Integrated cancer tissue engineering models for precision medicine

**DOI:** 10.1371/journal.pone.0216564

**Published:** 2019-05-10

**Authors:** Michael E. Bregenzer, Eric N. Horst, Pooja Mehta, Caymen M. Novak, Shreya Raghavan, Catherine S. Snyder, Geeta Mehta

**Affiliations:** 1 Department of Biomedical Engineering, University of Michigan, Ann Arbor, Michigan, United States of America; 2 Department of Materials Science and Engineering, University of Michigan, Ann Arbor, Michigan, United States of America; 3 Rogel Cancer Center, School of Medicine, University of Michigan, Ann Arbor, Michigan, United States of America; 4 Macromolecular Science and Engineering, University of Michigan, Ann Arbor, Michigan, United States of America; Monash University, AUSTRALIA

## Abstract

Tumors are not merely cancerous cells that undergo mindless proliferation. Rather, they are highly organized and interconnected organ systems. Tumor cells reside in complex microenvironments in which they are subjected to a variety of physical and chemical stimuli that influence cell behavior and ultimately the progression and maintenance of the tumor. As cancer bioengineers, it is our responsibility to create physiologic models that enable accurate understanding of the multi-dimensional structure, organization, and complex relationships in diverse tumor microenvironments. Such models can greatly expedite clinical discovery and translation by closely replicating the physiological conditions while maintaining high tunability and control of extrinsic factors. In this review, we discuss the current models that target key aspects of the tumor microenvironment and their role in cancer progression. In order to address sources of experimental variation and model limitations, we also make recommendations for methods to improve overall physiologic reproducibility, experimental repeatability, and rigor within the field. Improvements can be made through an enhanced emphasis on mathematical modeling, standardized *in vitro* model characterization, transparent reporting of methodologies, and designing experiments with physiological metrics. Taken together these considerations will enhance the relevance of *in vitro* tumor models, biological understanding, and accelerate treatment exploration ultimately leading to improved clinical outcomes. Moreover, the development of robust, user-friendly models that integrate important stimuli will allow for the in-depth study of tumors as they undergo progression from non-transformed primary cells to metastatic disease and facilitate translation to a wide variety of biological and clinical studies.

## Introduction

Tumors have long been viewed as the accumulation of a mass of aberrant cancer cells. However, research has repeatedly shown the dependence of cancer progression on a variety of environmental factors, including non-cancerous cells, mechanical stimuli, and the surrounding extracellular matrix (ECM), aptly naming it as a ‘cancer-organ’. Although many *in vitro* and computational models currently exist, the complex and interdependent microenvironmental regulation of the ‘cancer-organ’ system at the dynamic tissue and molecular scale have not been fully addressed.

Tumorigenesis and cancer formation is a complex multistep process involving genetic, epigenetic, and metabolic alterations, and interactions with the microenvironment that transform normal cells into malignant ones. As part of this process, oncogenes get activated, and tumor suppressor genes get repressed, affecting cell proliferation, apoptosis, pro-tumoral inflammation, avoiding immune surveillance and destruction, promoting genomic instability, angiogenesis, and metastasis[1,2].

As the tumors progress, new aberrant blood vessels continue to sprout due to activation of angiogenic switches in order to sustain proliferating malignant cells. The excessively proliferating autonomous neoplastic cells invade the local tissue, following which they intravasate into nearby blood and lymphatic vessels. Through these conduits, the disseminated cancer cells transit to distant organs, ultimately homing into specific niches after extravasating the blood/lymph vessel lumima. At the secondary sites, they form micrometastasis, which include small nodules of cancer cells, followed by growth of these lesions into macroscopic tumors, leading to metastatic colonization[1,2].

Due to diverse interactions involved, cancers are highly heterogeneous organ-like masses. Their complex microenvironments not only contain the tumor cells, but also various infiltrating endothelial, hematopoietic, stromal, immune and other cell types, ECM components, biophysical characteristics and mechanical stimuli [3–5]. Interactions within microenvironment also help create metabolic changes, such as a hypoxic environment and nutrient fluctuations, which further contribute to heterogeneity of cancer cells.

With this multifaceted network of communication between the native tissue and the tumor taken into consideration, cancer is more aptly understood as a complex organ, dependent on and working within the various colonized organs. This view of cancer provides a realistic perspective which allows us to increase our understanding of the disease, and thus identify crucial aspects for facilitating drug screening and development of efficacious, individualized cancer therapies.

**Fig 1:**
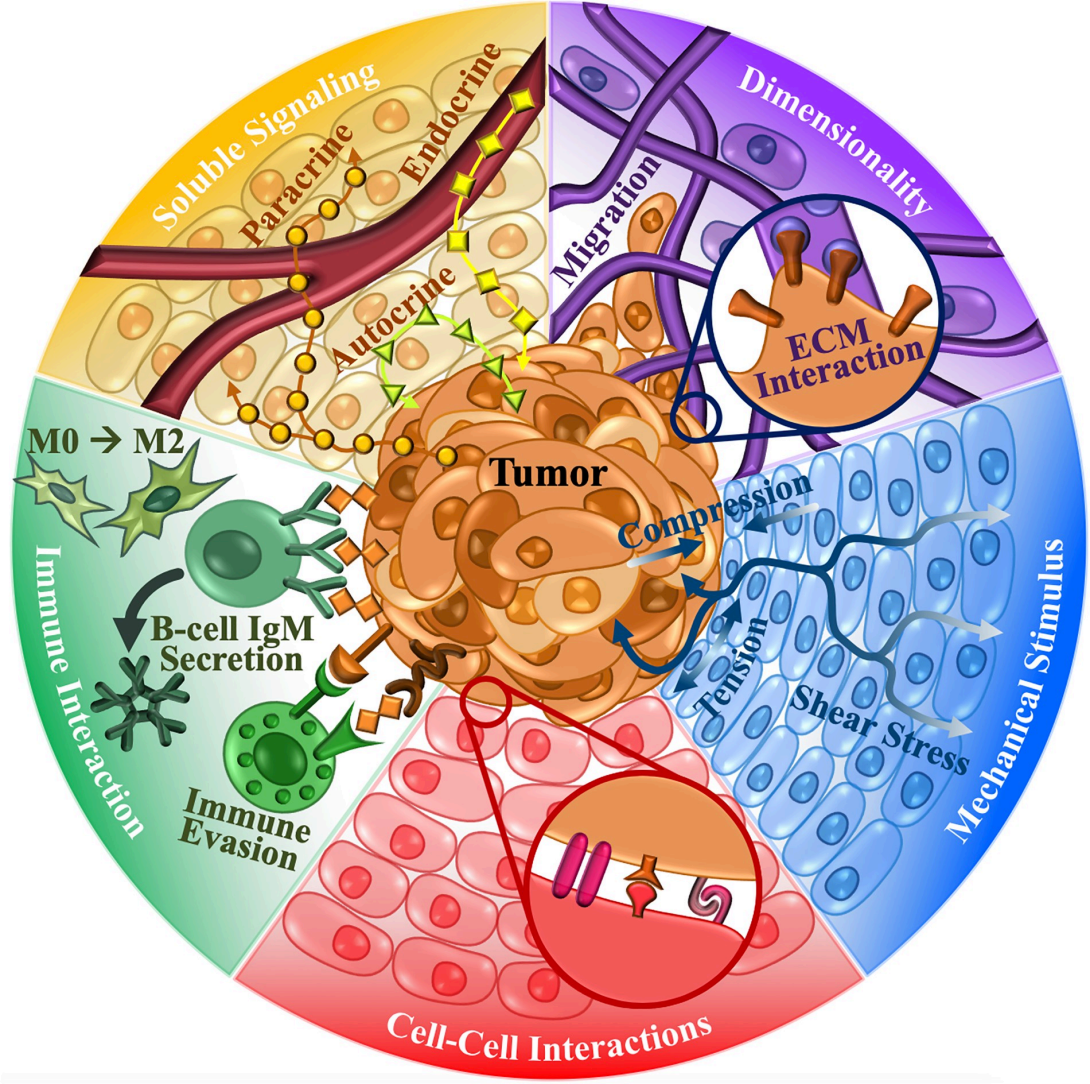
Components of the ‘Cancer-Organ’ model. To develop an accurate multi-dimensional understanding of the structure, organization, and complex relationships in cancers, we need to consider the following factors. Heterogeneous cancer cells reside in a complex tumor microenvironment, which consists of mechanical stimuli, non-malignant cell-cancer cell interactions, soluble signals, and extracellular matrix (ECM). The dimensionality of cell culture influences cancer cell motility and cellular interaction with the surrounding cells and ECM. Mechanical stimuli including shear, compressive, tensile, and viscoelastic forces, dynamically influence cancer cells as the tumor grows. Similarly, cellular interactions through direct contact with surrounding non-malignant cells and soluble signals alter communication and downstream signaling. Interactions between immune cells and cancerous cells are highly complex and can lead to immune evasion and support of tumor progression. All of these characteristics play an integral role in tumor progression and are critical to forming a complete picture of the ‘cancer-organ’ system.

Investigative approaches and interpretation of the ‘cancer-organ’ system heavily influences research conclusions. For example, the growth of cells on 2-dimensional (2D) surfaces versus 3-dimensional (3D) constructs alters a cancer cell’s response to chemotherapeutics, thus influencing drug development and perceived effectiveness[6]. Similarly, mechanical stimuli innate to the microenvironment and exacerbated by the growth and development of the tumor can alter the stemness of the cancer cells[7] along with metastatic tendencies[8–10]. Meanwhile, cellular interactions between the non-malignant cell populations, immune components[11,12], and cancer cells influence the advancement of the disease, as well as, the response to common treatments[13]. Additionally, acellular aspects of the microenvironment, including soluble signaling and ECM composition and architecture, play a large role in phenotypic behavior[14,15] and thus the conclusions of experimental outcomes. Each of these factors uniquely impacts cellular components within the tumor microenvironment (TME), contributing to the complexity of the ‘cancer-organ’ system (Fig 1). However, our in depth understanding of these factors and their complex interplay is limited by current model systems, which fail to corroborate findings and elicit sufficient reproducibility within the field.

**Fig 2:**
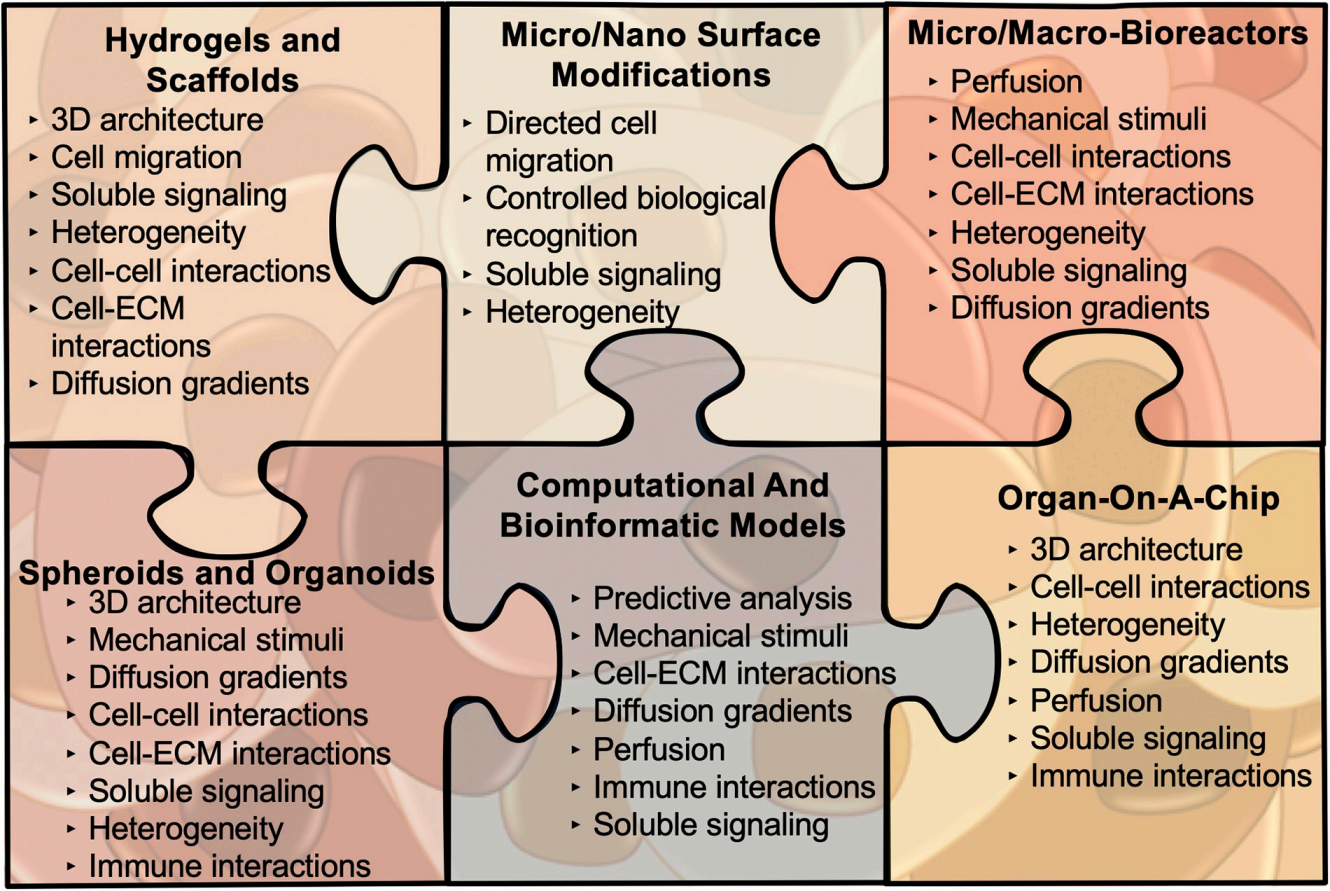
Various engineering tools can help construct the complex picture of the ‘cancer-organ’ system. Summarized here are the state-of-the-art cancer bioengineering models that we discuss in this review. Each model has inherent benefits and drawbacks that are discussed in more detail within the following sections. We have listed the components of the ‘cancer-organ’ system which can be probed with the specific model in the figure.

Therefore, the question remains, how do we as researchers reframe our understanding of cancer to encompass the many key players within the system. To address this dilemma, we have compiled a review of impactful cancer bioengineering models that investigate the important factors within the ‘cancer-organ’ system, including: dimensionality of cell culture (Section I), mechanical stimuli (Section II), multicellular interactions (Section III), immune interactions (Section IV), soluble signaling (Section V), complex cancer bioengineering models (Section VI), and mathematical models (Section VII), (Fig 1). These model systems portray the application of tissue engineering principles in understanding cancer biology and translating discoveries to delivery systems and precision medicine. We shed light on current state-of-the-art engineering methodologies that can help construct integrative cancer models in the first part of the review (Fig 2). In the latter half of the review, we provide suggestions of how to improve the quality and reproducibility of *in vitro* models and their findings (Section VIII), enabling the accelerated progress of cancer research as a whole. The ultimate goal of these integrated multi-scale models is not only to improve the understanding of cancer biology, but also to catalyze effective and personalized drug screening and therapeutic strategies that take the entire integrated complex TME into consideration.

## Dimensionality of the ‘Cancer-Organ’ models

Integrated ‘cancer-organ’ *in vitro* models are limited by the continued use of 2D cell culture, common due to ease of use. However, cancers share no similarity to cells grown in 2D, and as previously described above, grow as ‘organs’[3–5]. To improve the biologic relevance of ‘cancer-organ’ models, many studies have shown that 3D cultures are more reflective of the *in vivo* TME resulting in more physiologic cell behavior[6,16]. Specifically, compared to cells cultured in flat, rigid 2D culture dishes, cells in 3D cultures have different spatial arrangement of their surface receptors because they are surrounded by other cells and have 3D spatial constraints[17]. This 3D arrangement ultimately alters cell polarity, signal transduction, gene expression, cell morphology, growth rates, and other phenotypes[18–21]. 3D culture is also inherently more representative of the *in vivo* TME, as it allows for 3D nutrient and oxygen gradients, as compared to 2D culture, which results in a homogeneous distribution of nutrients and oxygen[22,23]. 2D cultures may also skew experimental results through unintentional selection of proliferating cells, as necrotic cells will not adhere to the tissue culture dish and will be removed during standard cell culture maintenance[23]. As a result of these differences, research has shown that drug response found in 3D models is more reflective of an *in vivo* response as compared to 2D cultures.

Consequently, growing number of cancer biologists and engineers alike are exploring 3D culture methods, as they have been shown to be more representative of the *in vivo* TME than traditional 2D methods[24–28]. For example, when comparing lung cancer cell growth and behavior on a polyester-based composite 3D scaffold versus 2D monolayer culture, it was found that the cancer cells had morphology more representative of *in vivo* tumors including necrotic centers, as well as upregulation of CD44 and carbonic anhydrase IX[29]. MCF10A breast cancer cells demonstrated changes in IL-6, H-Ras and E-Cadherin expression when grown in 3D within a Matrigel hydrogel, similar to those found *in vivo* [30]. Additionally, breast cancer cells were shown to have enhanced HER2 activation in 2D culture as well as a switch in signaling from phosphoinositide 3-kinase in 2D culture to mitogen-activated protein kinase in a 3D cell spheroid[31]. Drawing from these findings, it is clear the dimensionality of cancer cell culture greatly influences cell phenotypes, protein expression, drug response, precision medicine, and thus experimental results. The methods and materials chosen to formulate the 3D environment will interact uniquely with cell cultures, and these inherent benefits and drawbacks of each are discussed below.

The currently available 3D cell culture models include non-adherent suspension culture, hydrogels, 3D bioprinting, and scaffolds. Within each of these subtypes, material and fabrication variations can alter important culture parameters, such as cell adhesion, ECM structure, and ECM stiffness. In non-adherent 3D cultures, all possible cell attachment structures are removed as with rotary vessel/spinner flasks[32], hanging drop arrays[32,33], superhydrophobic surfaces[34], aqueous two-phase systems[35], and liquid overlays[36]. These culture methods usually result in the formation of spheroids, 3D microtissue aggregates of cells in the culture. These non-adherent models by their nature emphasize cell-cell interactions and are discussed in detail in Section III.I.

Another method of creating a 3D culture is to introduce an ECM-like element, such as a hydrogel or scaffold. These ECM mimics can fully encapsulate the cells within the matrix material, providing cells with structural, adhesive, mechanical and physical cues. Adherent hydrogels provide 3D support by allowing the cells to move and interact in all three (x, y and z) directions and interface with their environment on all surfaces of the cell as opposed to only laterally (x and y) in a 2D culture. Hydrogels also tend to emphasize cell-matrix interactions, which also include mechanical stimuli. Scaffolds are typically more rigid than hydrogels, and have micro- or macro-porous structures. They allow cells to migrate in 3D, interact with other cells in the construct, and provide a spacious microstructure that allows for cell-based ECM construction. Cell-matrix and cell-cell interactions are present due to the cell clusters that grow within the scaffolds. The chemical composition of the material chosen is crucial as it influences cellular response in terms of proliferation, migration, matrix rearrangement, and cell cluster formation[37,38]. For example, a biologically recognizable material such as collagen stimulates cell adhesion while cells do not interact with an inert material such as agarose[39,40], which acts as a blank background environment. Scaffolds and hydrogels can also be combined, creating a unique 3D environment that can both encapsulate cells while providing enhanced rigidity of the overall construct.

Of note, cells cultured in a 3D scaffold show more similarities to cells in human tumor tissues compared to 2D cultures. For example, glioblastoma U87 cells form round or ovoid shapes, and develop complex structures with cilia or microvilli when grown on a 3D collagen scaffold but exhibit an epithelioid morphology in 2D culture. These cells also exhibited therapeutic responses similar to responses seen in patients with glioblastoma[41]. Other properties of 3D cultures, such as stiffness and porosity, can also be adjusted through choosing or tuning an appropriate material or fabrication process to influence cell viability, migration capability, and flow of nutrients and waste in the culture[38]. A major limitation of most *in vitro* 3D culture systems is the lack of vasculature, which may affect our understanding of drug efficacy due to its role in drug, oxygen, and nutrient delivery. Though the 3D system does recreate cell-cell and cell-ECM interactions, it remains difficult to introduce physiologically relevant vascularization into these constructs, limiting the nutrient and waste exchange to the cell cultures, though some systems have been successful[42,43]. The challenge of vascularity models is further discussed in Sections V and VI.

The 3D culture method chosen for a given experiment should be chosen carefully based on the biological question being asked and on the inherent benefits and drawbacks, summarized in Table 1. Conscious design of 3D cultures with regard to these parameters is imperative due to their effects on cell behavior and overall effect of the ‘cancer-organ’ system, highlighting the need to develop model standards that most accurately replicate *in vivo* conditions. 3D models still currently lack a standardized reporting and characterization system. Without these standards, our understanding of the complexity of the ‘cancer-organ’ system falls short and creates the conditions for discrepancies between findings and lack of reproducibility. Suggestions for how to standardize reporting of 3D culture properties are discussed in section VII.II.

**Table 1:** Summary of 3-dimensional cancer bioengineering methods and their respective benefits and limitations.

	Material		Benefits	Drawbacks
**Synthetic Hydrogels**	Poly(ethylene glycol) (PEG)[6] Poly(vinyl alcohol)(PVA)[44] Poly(2-hydroxy ethyl methacrylate) (PHEMA)[45] Poly(acrylic acid)(PAA)[46] Poly(ethylene oxide)(PEO)[46]		Tunable stiffness Innately inert to cell adherence Highly reproducible Easy manufacturing Modifiable bio-functionality: growth factors, cleavage sights, tunable attachment motifs	Unreacted reagent impurities Dissimilar to biological materials
**Natural Hydrogels**[47]	**Inherently Interactive** Matrigel[48–50] Collagen[37,49,51] Gelatin[52,53] Hyaluronic acid[54] Chitosan[55]	**Inherently Inert** Alginate[56] Agarose[39] Dextran[57]	Biocompatible Low immune response Innate bioactive motifs	Batch-to-batch variability Poor mechanical tunability Extraneous bioactive signaling Ill-defined composition Irregular degradation rates Poor long-term stability *in vitro*
**Scaffolds**	Poly(lactide-co-glycolide) (PLGA)[58–60] Poly(caprolactone) (PCL)[61] Polystyrene[62]		Mechanical, chemical, and structural tunability Provides space for cell-based ECM synthesis	May require surface functionality to enable cell attachment Often requires organic solvent which are cytotoxic Difficult cell removal/imaging
**Suspension Cultures**	Ultra-low Attachment Plates[32,63]		Durable maintenance Isolated replicates High throughput	Supporting structure from well Expensive non-adherent coating Complicated surface coatings
	Hanging Drops[32,33,64,65]		Control of spheroid size Isolated replicates High throughput Medium only interface Imaging capability	Time intensive Skilled user Delicate maintenance
	Spinning Flasks/Nutators/Rotators[32,66]		Ease of use Autonomous Durable maintenance Batch based high throughput	Non-isolated replicates Inherent shear stress stimulation Uncontrolled spheroid size

## Mechanical Stimuli in the ‘Cancer-Organ’ models

The tumor microenvironment in the ‘cancer-organ’ induces mechanical tension, compression, and shear stress on the growing tumor, while exposing the cells to increased ECM stiffness and variable viscoelasticity. Therefore, these stimuli are explored in many mechanical cancer models[10,67–69]. For samples of micro an macro bioreactors utilized for examining mechanical stimuli in cancer, readers are referred to references [7,70–73]. As a wide variety of bioreactors exists to explore the dynamic mechano-environment a comprehensive list of devices is beyond the scope of this review. Over-proliferative tumor cells and an increase in interstitial fluid pressure, caused by tumor-initiated angiogenesis, orchestrate circumferential mechanical stretching along the tumor’s leading edge. To study this phenomenon, microreactors with flexible membranes are often used to stimulate stretching within the TME. Stretching using the microreactor models has been shown to promote cancer cell growth and induce proliferation[70,74,75], as well as upregulation of the YAP/TAZ pathways[74]. Commercially available bioreactors provide varying levels of uniaxial or equiaxial tensile force, and can be applied to ‘cancer-organ’ models[76].

The surrounding tissue provides resistance to the expanding tumor. As a consequence, the tumor is exposed to high levels of solid stress and the cancer cells experience ever increasing compressive force[77–79]. Scaffolds to study cancer compressive mechanotransduction have been fabricated using poly(lactide-co-glycolide) or hyaluronic acid, seeded with cells, and exposed to cyclic loading via compression bioreactors[80,81]. These bioreactors are typically designed and built in-house to allow for fine-tuned compressive loading cycles, although commercial options do exist. As a variation to this approach, a hydrogel can be embedded with cells and exposed to static compression by using a weight or piston to achieve the desired force[9,73,82,83].

The role of TME stiffness on cancer phenotype has been studied with a variety of models. For example, surface functionalized PDMS microposts have been engineered for specific stiffness and used to evaluate individual cell mechanics and protein expressions[84]. In another approach, increasing the polymer concentration of hydrogels during fabrication also modulates the hydrogel’s stiffness. Employing this technique allows the effects of stiffness to be tested on cancer cells without changing the substrate to which cells adhere[71,85–87]. Optical tweezers have also been used to study the effects of stiffness of single cancer cells[88]. Dynamic ECM models that replicate the ECM remodeling during cancer progression to support tumor growth, are increasingly becoming popular, since they modulate physical properties over time[89].

Most human tissue and polymer or protein ECM analogues experience varying degrees of an elastic strain during loading cycles. These viscoelastic matrices show time dependent recovery when loads are removed. In recent years, the viscoelastic nature of the TME has been shown to impact tumor matrix remodeling in collagen, fibrin, alginate, reconstituted basement membrane, and agarose hydrogel models[90]. Viscoelasticity has also been shown to impact cancer cell invasion in interpenetrating network hydrogels with low molecular weight RGD-alginate and reconstituted basement membrane, as well as collagen type I hydrogels[91,92]. Given the importance of ECM viscoelasticity in cancer progression and metastasis, additional studies are required to model and probe viscoelastic changes in the TME.

In addition, the TME is under a constant barrage of fluid-induced shear stress. Leaky vasculature within the tumor niche as well as venous blood flow has been shown to exert shear stresses ranging from 0.5 to 4.0 dyn/cm^2^. Circulating tumor cells and metastatic cells undergoing intravasation and extravasation may also experience a range of arterial shear stress from 4 to 30 dyn/cm^2^[93]. Shear stress is often tested using a microfluidic device design where growth medium is pumped through the closed system using a syringe or circulating pump. A narrowing of the flow channel within the device allows for pronounced wall shear stress and controlled laminar flow. We refer the reader to the review by Huo et al. for other forms of microfluidic devices implemented in the field of cancer mechanobiology[94].

Shear stress stimulation has been shown to increase proliferation[72], upregulate the pro-survival ERK pathways[95], enhance motility via YAP/TAZ[96], and increase chemoresistance[7]. Parallel plate[97] and rotary bioreactors[98] that apply shear stress to cells have also been used to study adhesion mechanics of tumor cells. Perfusion bioreactors typically provide cancer cells with relatively uniform shear stress across the entirety of the polymer scaffold. The TME may also be fine-tuned by manipulating the composition of the polymer or hydrogel. Taken together, there is ample evidence of the significant role that the mechanical forces in the TME play in tumor progression and of the variability present between models used to study mechanical stimuli. Importantly, many bioengineered models fail to consider mechanical stimuli all together, potentially resulting in unrealistic results. It is clear that mechanical stimuli influence key hallmarks of cancer and that the tumor requires this stimulus to elicit specific functionality; however, how cellular processes sense each of these stresses and translate them to oncogene expression is still poorly understood.

## Multicellular interactions in the ‘Cancer-Organ’ models

**Fig 3:**
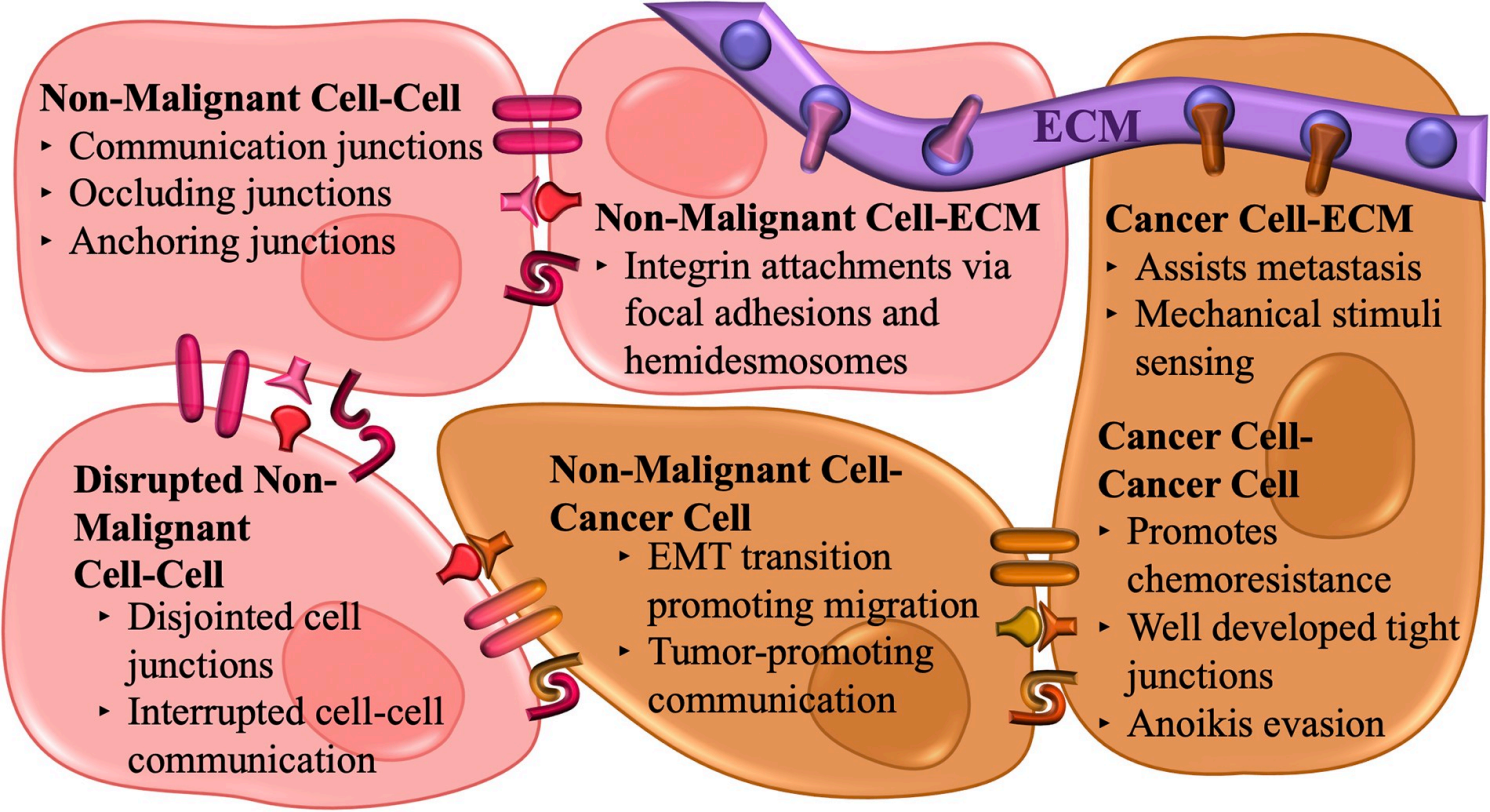
Various cell-cell interactions within the cancer-organ system. Interactions of cancer and malignant cells with their surroundings help dictate their survival and phenotypes. Within the homeostatic non-transformed microenvironment, various cell-cell junctions are formed ensuring the proper polarization, orientation, and proliferation of the non-malignant cells. Cell-ECM interactions provide structure and mechanical stimuli to the cellular surroundings through points of adhesion. These native interactions are disrupted by the infiltrating cancer cells which interrupt cell-cell communications and displace healthy tissue. The cancer cells undergo the epithelial-mesenchymal transition in order to metastasize and do not experience the same proliferative inhibition provided by non-malignant cell-cell communication. Well-established communication between cancerous cells increases survival by avoiding anoikis and promoting chemoresistance. Finally, the surrounding ECM, which is stiffened by the presence of the expanding cancer mass, aids in additional cancer cell migration, and an altered mechanical environment will feed forward the progression of the disease.

Cells and ECM come together to form tissues, which then collectively form structurally stable and functional organs. In contrast, tumors are a non-random mix of cells and ECM but are functionally and structurally unstable and abnormal. The cues from the TME non-cancerous cell types (including immune cells, endothelial cells, fibroblasts, and mesenchymal stem cells, among many others) have been shown to be instrumental in tumor initiation, progression, and metastasis[99–102]. In this section and in Fig 3, we describe some of the important modes of cell-cell communication components and their roles in the TME and ‘cancer-organ’ models.

Cells within the TME communicate via soluble signals, as well as, through physical connections, such as communication junctions (connexins, ion channels, chemical synapses, and pannexins), occluding junctions (tight junctions), and anchoring junctions (adherens, desmosomes, focal adhesions, and hemidesmosomes)[72]. These physical cell-cell communication components, which comprise specialized intercellular junctional proteins, are critical for maintaining cell polarity, barrier function, morphogenesis, differentiation, homeostasis, cell growth, and cell-cell interactions. All of these junctional complexes are used by cancer cells to transmit signals to the neighboring cells, as well as, to respond cohesively to various conditions. Furthermore, integrins facilitate cell-ECM interactions via focal adhesions and hemidesmosomes[103–107], allowing for cells to communicate with their physical surroundings.

These junctions are often disrupted in cancers via genetic mutation or epigenetic changes ultimately affecting tumor progression[108]. For example, tight junctions create a barrier in endothelial cells, allowing molecules and inflammatory cells to pass, whereas within epithelial cells, tight junctions work in an adhesive manner, keeping cells correctly polarized and preventing cells from distortion[109,110]. During intravasation, cancer cells distort the tight junctions of the vascular endothelium to penetrate it, which is considered one of the most critical steps in the dissemination of cancer cells. Thus, the tight junctions are the initial barriers the cancer cells must overcome in order to metastasize[111,112]. Similarly, an important step in metastasis is the epithelial-to-mesenchymal transition (EMT), which involves a cadherin switch from E-cadherin to N-cadherin promoting the migratory capacity of the tumor cells[108].

Additionally, previous studies have indicated that desmosomes undergo transformation during cancer progression[113]. Studies have shown that desmosomal proteins have both tumor-promoting[114] and tumor-suppressive[113] functions in different types of cancers. In particular, desmocollin1 (DSC1) and desmocollin3 (DSC3) may act as prognostic markers for lung cancer[115], colorectal cancer[113] and in esophageal, head and neck cancers[116]. As a major component of sensing the mechanical environment and the surrounding cells, proteins involved in cell adhesion are crucial aspects of tumor development. Cancer bioengineering models need to mimic these physiologic cell-cell and cell-matrix adhesions, interactions, and modifications in cancer to remain true to the complexity of cancer. A summary of these interactions is depicted in Fig 3.

### 3.1 ‘Cancer-Organ’ models emphasizing direct cell-cell interactions

Among the currently available 3D physiological integrated models of cancers, the following prioritize cell-cell interactions: 1) multicellular tumor spheroids; 2) tumorospheres; 3) tissue-derived tumor spheres; 4) organotypic multicellular spheroids[117,118] and; 5) organoids[119,120]. We described these 3D models in Section I. Others include microfluidics, aqueous two-phase system, and microfabricated microwell array[118,121]. The following section details the features of and differences between these 3D cancer models. Table 2 highlights some of these examples.

**Table 2:** Examples of 3-dimensional cancer bioengineering models that emphasize cell-cell interactions.

Model	Examples
Multicellular tumor spheroids	MCF-7 breast cancer MCTS formed in 96-well plates coated with 1% w/v agar and liquid overlay technique[36]
	MCF-7 breast cancer MCTS formed in chitosan-collagen-alginate scaffold formed with spray-spinning[240]
	Multicellular gastric spheroids formed via liquid overlay technique in 24-well plates coated with 1% SeaPlaque agarose diluted in serum-free RPMI-1640 medium[123]
	HCT116 colon cancer MCTS formed in a 96-well plate on top of a layer of agarose[241]
	HepG2 liver spheroids were formed in mixtures of 1:1 Matrigel:medium, gelatin type A from porcine skin, or collagen type I[242]
	Ovarian cancer cell lines A2780 and OVCAR3 were used to form spheroids in 384-well hanging drop plates with 10, 20, 50, or 100 cells per well[65]
Tumorospheres	MCF-7 Breast Cancer Tumorospheres formed in low attachment plates[243]
	Lung cancer tumorospheres formed in ultra-low attachment plates[244]
	Small cell lung cancer tumorospheres formed via collection of circulating tumor cells from blood and culture in normal tissue culture conditions[245]
	EGFR-mutant HCC827 and EGFR wild-type A549 cell lines cultured in low-attachment 6-well plates[246]
	HCT116 and HT29 colorectal cancer cell lines cultured in low-attachment 6-well plates[247]
	Human and murine derived prostate cancer cell lines plated in 12-well plate with a 1:1 medium to Matrigel ratio[248]
Tissue-derived tumorospheres	Prostate cancer LuCaP cells derived from primary and metastatic human prostate cancer xenografts formed spheroids in 6-well ultra-low attachment plates[249]
	Primary colorectal cancer cells were harvested with mechanical and enzymatic digestion to form spheroids with the cell clumps that maintained their cell-cell contacts[250]
	Tumorospheres were formed from a recurrent pineoblastoma tumor following mechanical dissociation in serum free medium[251]
Organotypic multicellular spheroids	Human colorectal cancer tissue resections were cut, minced, and incubated in agar coated tissue culture flasks to form organotypic multicellular spheroids[133]
	Fresh human and murine tumor specimens were minced, digested, and filtered prior to culture in ultra-low attachment plates. Spheroids were then mixed with type I collagen hydrogels and injected into a 3D microfluidic device[252]
	Inflammatory breast cancer cells were used to form PDX tumors which were subsequently harvested and used to form organotypic spheroids using the Bio-AssemblerTM (Nan03D Biosciences,Inc.) system[253]
Organoids with Stroma**l** Component	Human colon cancer cells were used to form multicellular spheroids followed by culture with normal fibroblasts or cancer-associated fibroblasts in collagen type I hydrogels[254]
	Co-culture of melanoma cell lines with vascular endothelial cells in 2D monolayers and 3D spheroids in 96-well round bottom culture plates with methyl-cellulose[255]
	Non-small cell lung cancer cell lines A549 and Colo699 cells were cultured alone or with lung fibroblasts within automation compatible hanging drop plates[256]
Organoids with Immune Component	Pancreatic cancer cell lines and primary cells were co-cultured with T lymphocytes or patient-matched fibroblasts in matrigel drops in 24-well culture dishes[257]
	Primary tumor-derived colorectal cancer organoids co-cultured with IL-2 starved cytotoxic T cells in basement membrane extract within 12-well culture dishes[258]
	Lymphocytes isolated from the small intestine were co-cultured with intestinal stem cell derived organoids in Matrigel[259]
Organ-on-a-chip	Heart-on-a-chip composed of a mechanically tunable poly(octamethylene maleate (anhydride) citrate) matrix surrounding a 3D microchannel vascular network lined with endothelial cells[260]
	Heart-on-a-chip composed of human induced pluripotent stem cell-derived cardiomyocytes in fibrin gel within a PDMS microfluidic device capable of applying cyclic strain[261]
	Liver-on-a-chip polymethyl methacrylate bioreactor capable of real-time glucose, lactate, and oxygen sensing seeded with growth arrested HepG2 liver cells and oxygen sensitive probes[262]
	Bone-on-a-chip PDMS device designed with a top medium layer separated from a culture chamber by a dialysis membrane. The culture chamber was seeded with osteoblasts in collagen-forming medium to form mature osteoblastic tissue[263]
	Lung-on-a-chip device made in PDMS with a compartmentalized 3D microchannel divided into two culture compartments by a microporous membrane of PDMS. Alveolar epithelial cells and pulmonary microvascular endothelial cells were seeded into the top and bottom culture chambers respectively[264]
	Gut-on-a-chip made in a microdevice separated by a porous PDMS membrane coated with type I rat tail collagen and Matrigel for adherence of human intestinal epithelial (Caco-2) cells[265]
Tumor-on-a-chip	A vascularized microtumor model in a polydimethylsiloxane (PDMS) microfluidic device with endothelial cells self-assembled into interconnected networks with luminal flow. Human colorectal cancer cells were added into the tissue chambers to form spheroids[266]
	Metastasis- and Bone-on-a-chip PDMS device designed with a top medium layer separated from a culture chamber by a dialysis membrane. The culture chamber was seeded with osteoblasts in collagen-forming medium to form osteoblastic tissue. Metastatic breast cancer cells (MDA-MB-231 cells) were seeded into the osteoblastic tissue region to study formation of a metastatic niche[263]
	PC-3 prostate cancer cell line cultured in 3D spheroids with endothelial cells and osteoblasts within a 2-layer microfluidic system to model bone metastasis[267]

Multicellular tumor spheroids are generated from suspensions of single cells of immortalized cell lines in the presence of serum in non-adherent conditions[64,65,121,122]. Originally formed with just cancer cells, multicellular tumor spheroids have more recently been cultured with combinations of cancer cells with immune cells, fibroblasts, and endothelial cells to study heterogeneous interactions in the tumor tissue. These spheroids can vary in diameter from 100’s of microns to 3mm, with a degree of compaction depending on the cell line of origin and culture method[32,123–125]. At diameters larger than 400μm, spheroids can have an inner core of hypoxic quiescent cells and an outer layer of proliferating cells replicating the hypoxic pockets that can form within tumors[117]. They can also replicate the differentiation of the parent tumor and have been demonstrated to enrich for cancer stem-like cells[126]. Studies have shown multicellular tumor spheroids are more physiologically representative and display similarities to patient tumors in not only their proliferative index but also to cell morphology, cell–cell junctions, and ERK1/2, MAPK, and PI3K, AKT pathway activation[127].

Patient tumor-derived tumorospheres are obtained from fine slicing and partial dissociation of cancer tissue. These tumorospheres represent the histological features, gene expression profiles, mutations, and tumorigenicity of the parent tissue. Because tissue-derived tumorospheres are formed due to dissociation, they are exclusively composed of cancer cells[123–125,127]. Researchers have shown that E-cadherin is involved in cell-cell interactions in tissue-derived tumorospheres and E-cadherin/β-catenin complexes were shown to be tethered to the cytoskeleton. This organization has been demonstrated to strengthen cell–cell adhesion in other systems, suggesting that tissue-derived tumorospheres have strong inter- and intra-cellular interactions[124,127,128]. Tumorospheres can be cultured under serum free conditions from suspensions of single cells that are sorted from a population of cancer cells[128–130]. Single cells then expand clonally to produce tumorospheres. Due to the capacity of stem cells to survive in serum free conditions and expand clonally, tumorospheres are specifically suited to cancer stem-like cells and their enrichment. In fact, mammosphere or neurosphere formation from a single cancer-initiating cell plated in suspension is a gold standard for tumor initiation research. While tumorospheres do not fully replicate the TME, they have been useful in understanding cancer stem-like cells[117].

Organotypic multicellular spheroids arise from slicing tumor tissue fragments into sub millimeter pieces followed by culturing in a non–adherent system with serum and other supplements[131–133]. These spheroids are circular structures that can be frozen or cultured. Spheroids isolated from ovarian carcinoma ascites fluid are a special case in the organotypic multicellular spheroids family because unlike the other organotypic multicellular spheroids, they are not generated after tissue processing but are isolated directly from patient effusions. In addition to recapitulating the original heterogeneity of the tumor, these ovarian spheroids also maintain their stromal component[117,131]. For example, organotypic multicellular spheroids maintain the presence of macrophages and preserve vessels with striated fibers of collagen in association with fibroblasts that surround vascular elements[131]. In another example, organotypic multicellular spheroids from bladder cancer display cell cycle distribution, which is similar to that observed in original tumors[134]. For further analysis of the advantages and disadvantages of these four model types, we refer readers to a more thorough review by Weiswald *et al*.[117].

Organoids can be grown by embedding embryonic stem cells (ESCs), induced pluripotent stem cells (iPSCs), somatic stem cells, and cancer cells into a 3D matrix and letting the cells self-organize into ‘mini organs’ similar to the organ of origin in a serum free medium[120,135,136]. Apart from a 3D matrix, which acts as a substitute for ECM, most organoid cultures medium requires different growth factors to grow depending on the tissue of origin[137]. Organoids can be passaged serially every 1–2 weeks[136] and can be genetically manipulated with comparatively more ease than other cancer models[138,139]. Organoids have been formed with both healthy tissues, including kidney[140,141], lung[142], liver[143,144], brain[145], colon[146–149] and from tumors derived tissues, such as those formed from breast cancer[137], prostate cancer[150], glioblastoma[151], pancreatic cancer[144,152–154], liver cancer[144], and colon/colorectal cancer[146,155]. This biorepository of healthy and tumor-derived organoids is an extremely useful tool in studying drug screening, cancer development, and disease modeling and precision medicine[136].

Since cancer operates and presents as a complex organ, there are still gaps in knowledge that these models have not been able to fill despite their improved physiological relevance over traditional models. Although they have been made from multiple cancer types and have excellent reproducibility, they are not complete representations of the *in vivo* system, as most lack vascular networks and intact immune components. Additionally, these models do not allow for fine manipulation of ECM, as many of these models are non-adherent cultures, and the models that utilize Matrigel are subject to batch-to-batch variation. While addition of ECM substitutes, like Matrigel, provide for the lack of a basement membrane and may make the model more physiologically relevant, they may also incorporate undefined extrinsic factors[156] that may cause artificial experimental outcomes. Additionally, some of these spheroid models enrich for a rare population of cancer cells termed cancer stem-like cells but do not include surrounding non-malignant cells, thus preventing us from learning about the interactions of cancer stem-like cells with neighboring cells in their 3D niche. Furthermore, organoids derived from stem cells may be difficult to standardize due to the need for complex addition of soluble signals at precise temporal intervals to direct differentiation down the desired lineage[157]. Finally, the variability present in spheroid-based modeling techniques represents a barrier to consistency in cancer research findings, as the differences between each spheroid generation method can result in discrepancies in research findings[158]. Therefore, there is a need for more comprehensive models that incorporate the complex environment of cancer and better recapitulate cancer–cell communication and functioning of the ‘cancer-organ’.

### 3.2 Modulation of ratio and density of cells in ‘Cancer-Organ’ models

Regardless of the model utilized, the type and number of cells added to the ‘cancer-organ’ may vary depending on the stage and progression of cancer being studied. The ratio between differing cell types should also be considered to reflect the *in vivo* TME setting, considering the proliferation rate of each cell type and their properties when cultured *in vitro*[159]. In an example of this consideration, Eder et al. used the hanging drop method with a co-culture of prostate cancer cells and cancer-associated fibroblasts. They showed an increased number of cancer cells compared to fibroblasts within the spheroids, which reflects *in vivo* observations[160]. While non-cancerous cells initiate cell cycle arrest to stop proliferation, cancer cells proliferate indefinitely and display no contact inhibition. Expectedly, in a heterogenous 3D model of cancer, non-cancerous cells are subject to contact inhibition while cancer cells are not, thereby changing the ratio between cancer and non-cancerous cells and overall cell density[159]. Diffusion and exchange of soluble factors within cell culture is also directly influenced by heterogeneous cell density, emphasizing the need for spatial control of cell seeding. While most models do not allow for spatial control of cell seeding densities, 3D bioprinting methods for cancer cell patterning have recently been developed, including valve-based printing, laser based printing, and thermal and piezoelectric inkjet printing, which provide reproducible control over spatial distance between cell types (i.e., cancer and stromal cells)[161], theoretically enhancing reproducibility of model results. In summary, the seeding density, as well as, ratio between cell types needs to be carefully considered during the design of 3D integrated ‘cancer-organ’ models.

## Immune interactions in the ‘Cancer-Organ’ models

**Fig 4:**
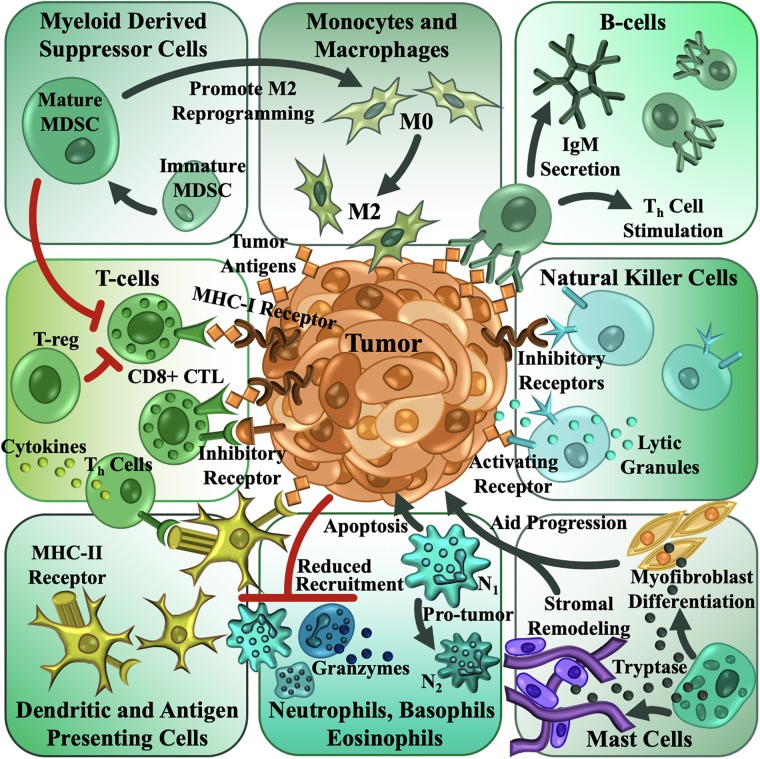
The immune microenvironment of tumors contains cellular components from both the innate and adaptive immune systems, with functional immuno-modulation between all the different cell types. Macrophages are typically the most abundant population of leukocytes within the TME, derived from both tissue-resident and circulating monocytic progenitors. The accumulation of tumor-associated macrophages is often correlated with the development of pathological phenotypes in cancer, which leads to the promotion of angiogenesis, metastasis, chemoresistance and functional suppression of adaptive immunity. The TME counterbalances activating natural killer (NK) cell signals with strong inhibitory signals to escape NK cell mediated immune surveillance and further reduce the phagocytic activity of NK cells. NK cells also exhibit functional anergic phenotypes with reduced phagocytosis and reduced amounts of cytoplasmic granules that contribute to tumor progression. Other granulocytes within the TME often recruited from circulating vasculature include neutrophils, basophils, eosinophils and mast cells. Tumors often experience reduced recruitment, but granulocytes are often re-programmed to a pro-tumor phenotype, promoting vascular normalization and stromal remodeling. Analysis of several solid tumors also indicate that they are infiltrated with T-cells and B-cells, recruited from circulating blood and lymphatic structures. The number of infiltrated T-cells offer significant prognostic value to cancers. However, the TME reprograms T-cells into an exhausted anergic state, leading to severe immune suppression, specifically of the Th and CTL (CD8+ cytotoxic T lymphocytes) phenotypes. Additionally, recruited naive T-cells are also converted to an insidious regulatory Treg phenotype, which contributes to suppressive immunomodulation. B-cells typically respond to tumor-derived antigens and elicit antibody responses through IgM secretion and direct stimulation of Th cells. Tumor-educated B-cells are immuno-suppressive, promote regulatory T-cells, and promote carcinogenesis. Myeloid derived suppressor cells are heterogeneous mixes of immature myeloid cells, found accumulated in lymphoid structures, blood, and the TME, and are heavily correlated with immune suppression. Myeloid derived suppressor cells are powerful inactivators of T-cells. Impaired myeloid differentiation also results in defective antigen presentation. Coupled with dysregulated T-cell priming by antigen presenters like dendritic cells, an overall immune suppressive landscape leads to tumor escape from immune surveillance.

The TME, although largely made up of neoplastic tumor cells, also contains stroma and several types of immune cells[162]. The immune contexture of solid tumors is very well described and reviewed for several different kinds of tumors[163,164]. Tumors feature infiltrating lymphocytes, mature and immature myeloid cells capable of differentiation, macrophages, dendritic cells, eosinophils, mast cells, natural killer cells, and myeloid derived suppressor cells[162,164]. The type, location, and density of immune cells within the tumor are considered valuable prognostic tools in the treatment of neoplastic malignancies[165–168]. The general consensus for immune cells within the TME is that their dysregulated and somewhat functionally impaired phenotype results in an immunosuppressive TME, allowing for tumor progression[169–173]. Alternatively, immune cells, such as activated macrophages in the TME, also play more direct roles in promoting angiogenesis and tumor cell migration and survival, leading to not just an immunosuppressive program but an overall thriving and conducive environment for tumor progression[169–173]. In the cancer immunosurveillance paradigm, neoplasia is largely controlled in its initial stages by various immune cells, so much so that immune cells were clearly demonstrated to perform tumor-specific rejection in transplanted tumors in mice[174,175]. The crux of this hypothesis was the new antigenic properties of tumors elicited a potent immune response, leading to tumor regression. In fact, in our current understanding of immunologic escape by tumors despite immune surveillance, tumor variants with reduced immunogenicity inherently cull their high immunogenic counterparts, leading to tumor progression. Today, this concept is broadly described as ‘cancer immunoediting’ with a range of actions from anti- to pro-tumoral scenarios. The classic immune surveillance paradigm falls into the ‘Elimination’ phase of immunoediting. An equilibrium process is presented where low immunogenic tumor variants are selected. Lastly, an escape process is reached where the tumor actively suppresses and creates anergic versions of immune cells within the TME, thereby promoting tumor tolerance and tumor progression[176–179]. A summary of the role of immune cells and their roles in the TME can be found in Fig 4.

Until recently, most bioengineering models provided little insight into the role of the immune cells in the TME, with cancer research dominated by immunosuppressive *in vivo* models and mono-culture *in vitro* systems[180–183]. Due to the important role that the immune cells can play in cancer progression, recent models have attempted to mimic the native immune component of the TME[184–186]. Some of these models take advantage of patient’s peripheral blood mononuclear cells from which dendritic cells, lymphocytes, monocytes, or natural killer cells can be derived[187]. This is an important source of immune cells, as the derived cells will be representative of that specific patient’s immune system.

Outside of generating biomaterial strategies and testing on murine models, *in vitro* models also afford an easy platform for testing. *In vitro* systems are gaining increasing importance in the era of personalized medicine, where tumor antigens from the cancer genome will be identified on a personalized basis using deep genome sequencing techniques[188–192]. For example, Herter *et al*. developed a heterotypic spheroid model, including tumor cells, fibroblasts, and immune cells. This system visualized immune cell infiltration and specific elimination of tumor cells upon immune cell activation with a novel immunocytokine IgG-IL2v and tumor or fibroblast targeted T-cell bispecific antibodies [193]. Another group created tumoroid and tumor slice cultures from patient-derived peripheral and tumor immune populations. This model demonstrated sensitivity and resistance to 5-fluorouracil and Lonsurf in the context of a heterogeneous tumor immune microenvironment[194]. The biggest advantage of these methods lies in the rapid establishment of the model system, which can be developed within weeks of surgical resection and provide personalized screening in the context of a functional immune system. The 3D architecture of such cancer/immune models helps to predict immunocytes interactions with tumor cells with higher fidelity. Indeed, culture of melanoma cells in 3D systems demonstrated impaired immuno-recognition by cytotoxic T lymphocytes when compared to 2D cultures[195]. Similarly, incorporating macrophages into 3D organotypic cultures of squamous cell carcinoma demonstrated a potent immuno-suppressive program, activating macrophages into a pro-tumoral alternatively activated phenotype with no external cytokine stimulation[196]. The inclusion of immune components in bioengineered *in vitro* models make them more representative of the *in vivo* tumor and will allow for improved cancer research and personalized treatments.

Recent progress in tumor immunology and identifying pharmaceutical targets focused on immunologic approaches have significantly moved the cancer immunotherapy field forward. While the idea of using immune tissue engineering to incorporate an immune component to cancer engineering has fundamental appeal, the immune system is a complex mesh of innate and adaptive immunity, with extensive functional immunomodulation between the two compartments. Facilitating this immunomodulation requires incorporation of several different types of immune cells, and sourcing these immune cells, whether from peripheral blood or tissue/tumor resident approaches, still requires better isolation procedures[4,197–199]. Furthermore, a fundamental understanding of how the TME tips the balance of maintaining peripheral tolerance while suppressing adaptive immunity is imperative in engineering the immune tumor component.

Development of better engineered tumor-immune models will also accelerate the progression and impact of the emerging field of cellular immunotherapies. A big component of cellular immunotherapies began with the advent of genetically engineered T-cells with T-cell receptors engineered to direct their cytotoxic activity toward tumor cells. Chimeric Antigen Receptor T-cells (or CAR T-cells) are an emerging cellular immunotherapy paradigm to treat both solid and hematogenous tumors[200–202]. CARs contain antigen recognition regions specifically directed against tumor-derived antigens or neo-antigens and intracellular domains that are combinations of co-stimulatory peptides. Engineered platforms can serve as wonderful tools to test and assess toxicity to mitigate adverse toxicity reactions reported in clinical trials for adoptive T-cell therapies[203,204]. For detailed reviews and perspectives on preclinical studies and early phase clinical trials in CAR T-cell therapy, readers are recommended to refer to Grigor et al. [205], Newick et al. [201], Mata et al. [206] and Yu et al. [207].

Other major challenges include the information not currently gained from a tumor’s genetic signature, including specifically the response of host immune cells within the TME. Several advances in enabling technologies are required for a high fidelity immune tissue engineering model that can replicate the immune complexity and response of the tumor when viewed as an organ. The following technologies in our opinion are of high value to translate tumor immune microenvironment engineering into patient-specific therapeutic strategies: rapid identification of the immune TME during diagnosis both locally within the tumor and peripherally incorporated from reliable clinical assays; integrated systems-based immuno-modeling approaches that could be used to develop predictive models based on individual immune cell behaviors[208–210]; and microfluidics or small-cell based approaches to understand individual immune cell interactions (e.g. T-cell-macrophage, or T-cell-T-cell, etc.) to be integrated into a whole ‘cancer-organ’ perspective[211,212].

## Soluble signaling in the ‘Cancer-Organ’ models

Just as soluble signals play an integral role in organ function and development[157], they are also critical in tumor development. In addition to direct cell-cell communication discussed earlier, cells within the TME also communicate through indirect means via soluble signaling and endocrine signaling[99–101,103]. These soluble signals include growth factors[213,214], chemokines[214,215], cytokines[214,215], exosomes[214,215], and hormones[216]. Importantly, cells in the TME also respond to chemical gradients, which act as another form of soluble signal. Specifically, oxygen levels have proven to be key determiners of tumor progression[217]. The composition of these signals in the TME affects critical aspects of a tumor, such as angiogenesis[104,218–220], proliferation[219], differentiation[221], drug response[220], and metastatic capacity[104,220,222]. In a tumor, soluble signal gradients can cause migration of immune cells[222], endothelial cells[219], and mesenchymal cells[223] to the tumor. These cells then act to either promote[224] or inhibit tumor growth[225]. Conversely, chemokine gradients may also contribute to the metastatic destination of tumor cells expressing the appropriate chemokine receptor[104]. Due to the complexity of soluble signaling in the TME, we will not discuss every facet in great detail, but rather we will highlight some key examples to further demonstrate the importance of soluble signaling in tumor progression and the models employed to study them.

A clear example of the profound effects that soluble signaling can have on tumor development is the effects of mesenchymal stem cell conditioned medium (MSC-CM) on tumor cell growth. A single dose of MSC-CM applied to SGC-7901 tumor cells resulted in tumor cell expression of VEGF and RhoA-GTPase and ERK1/2 activation leading to enhanced tumor growth that was maintained through serial transplantation experiments[224]. Furthermore, MSCs can differentiate into fibroblasts, which are typically responsible for synthesizing ECM components, maintaining tissue homeostasis, and regulating inflammation, proliferation, and differentiation in healthy tissue. In the presence of cancer cells, fibroblasts can be activated by cancer or immune secreted TGF-β, FGF-2, HGF, PDGF, and interleukins, as well as reactive oxygen species to become cancer-associated fibroblasts (CAFs)[223]. Activation of CAFs then results in an increased secretion of chemokines that promote tumor proliferation, invasion, and angiogenesis[223]. CAFs also interact with endothelial cells through secretion of FGF-2 and SDF-1. These factors promote angiogenesis and recruitment of endothelial cells respectively, which facilitate tumor growth and metastatic capability[219].

Additionally, long distance signaling with hormones (endocrine signaling) is an integral component of some cancers, so much so that therapeutics aiming to inhibit these signals have been developed[226,227]. For example, 17β-estrodiol (E2) is a hormone that affects the physiology of organs in males and females via binding to estrogen receptors. Upon receptor binding, E2 can trigger downstream signaling within the cell to regulate cell proliferation and gene transcription. Consequently, deregulated E2 can lead to the development of breast cancer and has been shown to determine the degree of breast cancer growth in 70% of all breast cancer cases[226]. Deregulated E2 can also cause increased cell migration and invasion[226]. As a result, inhibitors of E2 binding to estrogen receptors have been developed to treat breast cancers[226].

Oxygen gradients are also an important aspect of the TME. Due to rapid, uncontrolled proliferation, tumors have a tendency to outgrow their vasculature and grow faster than new blood vessels can form. This results in significant oxygen gradients leading to hypoxic pockets of cells. One of the major consequences of low oxygen levels is increased signaling via hypoxia-inducible factor (HIF), which results in the production of soluble signals that can affect epithelial-to-mesenchymal transition, angiogenesis, chemo- and radio-resistance, and metastasis, which are generally associated with poor clinical outcomes[217]. Furthermore, oxygen concentration has been shown to affect stem cell proportions within a tumor and select for radioresistant sub-populations[228] as well as the proliferative capacity of non-cancer cell types[219].

While soluble signaling can be evaluated in most bioengineered cancer models using techniques discussed below, a few models seek to isolate the effect of these soluble signals by preventing direct cell contacts between different cell types. For example, Regier et al. developed a compartmentalized system that allowed for tri-culture of breast cancer cells with stromal and immune components keeping each cell type physically separated while sharing the same culture medium. This allowed for direct analysis of paracrine signaling between co- and tri-cultures[229]. A similar model developed by Szot et al. was able to evaluate the role of paracrine signaling from breast cancer cells in causing angiogenesis of endothelial cells through culture of cancer cells and endothelial cells separated by an acellular type I collagen matrix[230]. It is important to note that models focused on paracrine signaling may yield unrepresentative results due to the absence of direct cell-cell signaling, though they are valuable for the evaluation of explicit paracrine effects.

Due to the pervasive effects of soluble signals in tumor progression and the dependence of soluble signaling on the culture model, soluble signals are often included in the analysis of cancer engineering models. However, the sub-micromolar concentrations of these signals within the TME complicates the design and capabilities of the models[221]. Typically, analysis occurs at the protein and/or RNA level using ELISA and RNA sequencing, respectively. Investigation of the effect of oxygen concentration requires cell culture in oxygen-controlled incubators and characterization of hypoxia using oxygen sensitive stains as well as analysis of protein and RNA level expression of hypoxia inducible factors. While methods of soluble signaling analysis are relatively uniform across labs, experimental variation is introduced by the wide variety of model systems implemented, which can affect the composition and concentration of soluble signals and thus differential cell behavior. This variation is caused by the incorporation of multiple cell types, culture dimensionality, mechanical stimuli, and immune cells in different cancer tissue engineering models.

The main limitation in ‘cancer-organ’ models that study soluble signaling is inherent in our inability to temporally recapitulate the *in vivo* microenvironment. The complexity of the environment often means that changing or omitting one component in the system can lead to a cascade of unrealistic effects within the tumor model. For example, it is known that MSCs can induce tumor cell proliferation and expression of VEGF[224], which in turn triggers angiogenesis and endothelial cell proliferation[231]. In turn, endothelial cells communicate with the tumor cells to drive progression[232,233]. In addition to the changes in tumor cells induced by MSC and endothelial cell signaling, the increased proliferation will also alter nutrient and oxygen gradients as well as mechanical forces present due to the increased tumor size, further affecting cancer cell behavior[77,79,147]. Therefore, any model that does not contain both endothelial cells and MSCs will result in only partial tumor cell changes which may lead to false conclusions. The same could be said for the omission of any aspect of the TME. Therefore, it is necessary to improve the complexity of our ‘cancer-organ’ model systems to preserve biologic relevance.

Finally, a point of concern for current models is that traditional cell culture is performed at oxygen concentrations around 20%, while physiologic oxygen levels in the TME are between 4 and 10%, meaning that most culture models are in hyperoxic conditions[217]. While some microfluidic devices modulate oxygen tension to physiologic levels[234–236], other models of physiologic oxygen tension typically involve traditional cell culture in a multi-gas incubator with oxygen control[237,238]. These models operate under the assumption that the oxygen concentration that the cells sense is identical to that inside the incubator, while in reality it will depend on medium height and cell density[237,238]. The clear effect that oxygen concentrations have in the TME brings into question the reliability of *in vitro* cancer model experiments conducted at atmospheric oxygen levels as well as oxygen control incubators in the absence of cell level oxygen tension measurements. That said, hypoxic conditions overall have been well studied showing a clear influences on a variety of cellular mechanisms, such as promoting genetic instability, metastasis, invasiveness, and chemoresistance[239]. Thus, oxygen concentration levels should be of note and consideration for all ‘cancer-organ’ and TME investigations.

## Complex and integrated ‘Cancer-Organ’ models

The lack of complexity in many *in vitro* models has led to poor efficacy in drug screening applications, leading to post-approval drug withdrawals. This is due to poor understanding of systemic drug toxicity and mechanism of action in a whole organ and precision oncology context[268]. Organ-on-a-chip models seek to remedy this problem through the integration of tissue engineering and microfluidics to develop more complex models that better recapitulate organ function and drug response. Specifically, these models aim to reproduce tissue microenvironments in terms of tissue level, multicellular organization, and tissue specific functions. Unlike organoid or tumoroid models, which rely on complex self-assembly of cells into organ like structures, organ-on-a-chip models provide more control over spatial confinement and allow for the connection of multiple organ systems. Recently, Zhang et al. developed an organ-on-a-chip model with built-in vasculature and a biodegradable scaffold made with poly(octamethylene maleate (anhydride) citrate). This chip allowed for parenchymal assembly on the matrix, which surrounded 3D, perfusable microchannels coated with endothelial cells. The permeability in the vessel walls allowed for enhanced intercellular communication as well as extravasation of monocytes and endothelial cells. Using this model, the authors created functionalized hepatic and cardiac tissues, which could be used to study the effect of drugs delivered through the vasculature[260]. Organ-on-a-chip devices have also been successfully developed to mimic the heart[260,261], liver[262,269], bone[263], kidney[270], lung[264], and gut[265] and have even been expanded to body-on-a-chip devices that integrate multiple organ systems into a single device[268]. Body-on-a-chip devices allow for inter-organ migration and communication as well as evaluation of systemic drug toxicity[268]. Culturing multiple “organs” on a single device can also serve as an ideal *in vitro* model of metastasis from a tumor to another organ. In fact, ‘patient-on-a-chip’ and ‘cancer/tumor-on-a-chip’ models are now being introduced to further individualize therapies.

Skardal et al. demonstrated this concept with the development of a metastasis-on-a-chip platform to study the metastasis of colon cancer cells co-cultured with epithelial intestinal cells in a ‘hyaluronic acid-polyethylene glycol diacrylate (PEGDA)-gelatin’ based hydrogel gut structure to a similar hydrogel containing HepG2 liver cells downstream in the microfluidic device[271]. This system also enabled mechanical tunability of the hydrogels via polymerization with linear, 4- arm, or 8-arm PEGDA to examine the effect of tissue stiffness on metastasis, showing increased metastatic ability in softer hydrogels[271].

Tumor-on-a-chip devices, like Skardal’s device, more accurately replicate the complex ‘organ-like’ microenvironment, leading to improved drug development and more accurate screening results compared to simpler *in vitro* models[272]. In fact, tumor-on-a-chip devices can overcome one of the main limitations persistent in most *in vitro* models: the lack of functional vasculature. The vasculature is an important component of *in vitro* models, as it provides oxygen and nutrients, delivers drugs and immune cells to the tumor, and serves as a channel for tumor cell migration and metastasis[272]. The endothelial cells that make up the vasculature also communicate dynamically with the tumor to direct tumor phenotype and angiogenesis via notch and vascular endothelial growth factor signaling, for example[232,233]. Tumor-on-a-chip devices demonstrate remarkable potential in regard to incorporation of realistic vascular components to a tumor model by creating vascularized channels within microfluidic devices.

One such device was fabricated using a polydimethylsiloxane (PDMS) microfluidic device with two outer channels (one arteriole and the other venule) connected by three ‘tissue chambers’. The tissue chambers were injected with endothelial and stromal cells with extracellular matrix. After 5–7 days of culture, the endothelial cells self-assembled and formed networks interconnected with the arteriole and venule channels directing flow within the lumen of the vascular network. Incorporation of human colorectal cancer cells into the tissue chambers resulted in the formation of tumor spheroids, which localized near the vessels and sometimes ensconced a region of the vessels recapitulating vasculature running through a tumor *in vivo*. Using this model, the authors were able to compare the effects of potential anti-cancer drugs, Pazopanib, Sorafenib, and Vincristine, on angiogenesis, maintenance of the vascular networks, and tumor spheroid growth[266].

Other advantages of tumor-on-a-chip devices include replication of key aspects of the TME like tumor-stroma interactions, tumor-ECM interactions, tumor-chemokine interactions[273], tumor-immune interactions, and complex processes like epithelial-to-mesenchymal transition and specific steps of metastasis like intravasation and extravasation[274]. Hao et al. developed a bone-on-a-chip device for co-culture of metastatic MDA-MB-231 breast cancer cells with mineralized collagenous bone tissue. Using this model, the authors studied breast cancer metastasis to bone and observed characteristics of breast cancer bone colonization that had previously only been seen *in vivo*, including rapid invasion of cancer cells into the apical layer of mineralized tissue, invadopodia that extended into distant matrix, cancer cells forming lines, and subsequent alignment of collagen parallel to the lines of cancer cells[263].

Finally, organ and tumor-on-a-chip devices can facilitate real-time analysis of experimental variables within the device[268]. This advantage was demonstrated in a liver-on-a-chip device that allowed for real-time analysis of metabolic function using a computer controlled microfluidic switchboard to measure glucose and lactate[262]. The ability of these devices to capture the complex multi-cellular, ‘organ-like’ environment of a tumor and neighboring organs combined with easy integration with analysis techniques make ‘on-a-chip’ devices promising platforms for the future of cancer research.

## Mathematical modeling in the ‘Cancer-Organ’ models

The complexity of the TME makes perfect replication *in vitro* an unlikely feat; however, mathematical modeling can be used to help fill in gaps of knowledge left by incomplete models of complex ‘cancer-organs’. Specifically, mathematical modeling techniques can help fill in gaps in mechanistic understanding, indicate experiments that should be performed, and make personalized predictions of patient response to treatment[275,276]. This idea has led to the development of many mathematical models of cancer.

As we learn more about the complexity of the TME, the need for mathematical models becomes clearer, as accurate experimental models become more difficult to create. For example, the recent discovery of Tie2-expressing macrophages (TEMs), which influence tumor angiogenesis, vascular remodeling, and monocyte differentiation, added another macrophage phenotype into the system of macrophage interactions with the tumor[277]. To study macrophage interactions with the TME and the resulting tumor progression, a mathematical model was developed with M1, M2, and TEMs interacting with a metastatic lesion in a highly vascularized organ. The model included simulations of M1 release of nitric oxide, M2 release of general tumor growth factors, TEM secretion of angiopoietin-2 and IL-10, and evaluated their effects on tumor progression. This model revealed that TEM effects on tumor growth are irrelevant in the presence of M2 macrophages, suggesting that TEM targeting therapies would need to be administered in conjunction with M2-targeting therapies[277]. These findings provide insight into the weight of importance of TEMs vs M2 macrophages in tumor progression, which would be technically challenging to do via experimental means alone.

In another mathematical model, the authors sought to reconcile the development of resistance to epidermal growth factor receptor inhibitor in non-small cell lung cancer (NSCLC) with the changes in the TME. Specifically, they modeled the effect of glucose, oxygen, and drug concentrations on tumor evolution, with cell death and division rates dependent on the amount of available glucose, oxygen, and drug in a given region. In this model, cells with inherent drug resistance had different death and division rates, and drug sensitive cells had a small chance of developing resistance with each division. They found that compartments of their model with different oxygen, glucose, and drug concentrations had different predicted rebound times from drug treatment. They also found that the rebound following drug treatment varied depending on the mechanism of drug resistance and was independent of the number of resistant cells due to differential selective pressures in the different regions of the microenvironment[278]. This model helps to explain the role of tumor heterogeneity in the development of drug resistance and tumor recurrence and highlights the need to create more comprehensive experiments and mathematical models that take this heterogeneity into account.

Other more comprehensive models of the TME take into account multiple length scales to incorporate individual atomic events and cell interactions with discrete agent-based models, as well as bulk tumor growth, oxygen and nutrient diffusion, angiogenesis, and multi-organ processes, such as metastasis with continuous differential equation based models[240,279]. By merging multiple size and time scales, a more realistic depiction of the whole ‘cancer-organ’ can be obtained, resulting in higher model prediction accuracy[240]. While these hybrid models are more complex, advances in computation hardware has made them more feasible in recent years[240]. Despite the advantages that are inherent in the complexity of multiscale hybrid models, simpler models are still useful, as they are less computationally demanding and can still provide valuable biological insights.

The broad range of mathematical modeling complexity and techniques gives them almost unlimited potential in cancer research. Aside from the specific models discussed, many models have been created to examine almost any aspect of the TME including: tumor-immune interactions[277,280–282], soluble signaling[277,282], tumor initiation and mutation rates[283], metastasis[284,285], angiogenesis[279,286,287], ligand binding events[282], development of tumor heterogeneity[276], proliferation and growth[288,289], dormancy[281,289], recurrence[278], extracellular matrix interactions[240,279], and drug resistance[278,290]. For a more detailed discussion of mathematical modeling techniques used to model the TME, readers are referred to several more comprehensive reviews focused on mathematical models of cancer[240,275,276]. Using these models to complement our experimental systems has the potential to add additional layers of complexity that would be difficult to study experimentally, thus helping to expand our understanding of the TME from a complex ‘organ-like’ perspective. Unfortunately, the specialized skill set required to implement these models serves as a barrier to the uniform adoption of mathematical models to supplement experimental research.

While cancer research has made many discoveries about the factors that influence tumor progression and developed new and improved treatments, we are still a long way from understanding the entire ‘cancer-organ’ system. More importantly, our lack of a complete understanding of tumor progression has slowed the development of novel treatments and potential cures. In the next part of this review, we will discuss the main challenges facing the field and propose solutions for improving physiologic and experimental reproducibility, as well as, the integration of biology and engineering.

## Challenges and proposed solutions for developing physiologic ‘Cancer-Organ’ models

### 8.1 Physiological reproducibility

The inherent difficulty involved in building a functional ‘cancer-organ’ in the lab translates directly to *in vitro* tumor models by virtue of the similarities between the complex organization of an organ and the TME. Namely, it is immensely difficult to recapitulate the dimensionality, the mechanical environment, the cell-cell interactions, the soluble signals, and the immune compartment of the TME in a single model that is amenable to precise analysis. Each of these factors of the TME is integral in directing tumor progression as discussed above and thus should be considered in the design of experimental models. When many of these factors are not considered in a given model, there is an increased risk of producing unreliable results. Herein lies one of the main challenges facing cancer research: the lack of physiologically representative *in vitro* models due to the abundance of variables that exist within the complex, 3D, multicellular, organ-like TME. While a lot of progress has been made in terms of making more physiologic models, they are often still tailored to a specific research question and maintain reasonable simplicity via inclusion of only the necessary characteristics of the TME. For example, an investigation of the role of fibroblasts on cancer progression is likely to involve a 2- or 3-dimensional co-culture of fibroblasts with cancer cells. Such a co-culture neglects the other cell types within the TME, their cell-cell interactions and contributions to the soluble signaling milieu, and the mechanical stimuli. While it can be important to study one TME characteristic in isolation to understand its individual contributions to tumor biology, it is equally important to verify those findings in a more complex model with multiple TME characteristics. That said, a perfect replication of the organ-like TME of a tumor would be very technically challenging and would likely limit the reproducibility of experiments across labs. To overcome this limitation, cancer tissue-engineered models that present a slightly more comprehensive representation of the tumor while maintaining reasonable degrees of simplicity need to be developed to ensure that experimental results truly reflect the *in vivo* system.

It is also essential to develop cancer engineered models based on the different approaches required by solid tumors and liquid tumors to ensure that a model accurately reproduces *in vivo* physiology. Similar to solid tumor models, researchers have used cell lines and sophisticated animal models in addition to the more recent use of ex vivo models using hydrogels and scaffolds[291] in order to replicate the tumor microenvironment of liquid tumors. However, the characteristics of the microenvironment of liquid tumors, such as acute myeloid leukemia (AML), are significantly different from the microenvironment of solid tumors [292]. These differences need to be reflected in physiological models of liquid tumors compared to solid tumors to obtain realistic results. Therefore, selection of an appropriate personalized bioengineered model based on the type of cancer is fundamental for target discovery and precision oncology.

While the ideal model system would incorporate cellular diversity, dynamic mechanical forces, cell-cell and cell-ECM interactions, chemical gradients, and soluble signaling in a 3D microenvironment that is amenable to downstream analysis, we propose that researchers try to design their models to include at least three of the TME characteristics discussed in this review. Including additional TME characteristics into a culture model beyond the minimum necessary for the study will improve the robustness of the findings. If this is accomplished, our understanding of tumor progression will be greatly improved, and the identification of efficacious patient-specific treatments and treatment strategies will be accelerated. To best accomplish this, a multidisciplinary research approach involving collaborative efforts between engineers, oncologists, and immunologists is paramount to closely recapitulating the physiological TME and accelerating the race to cancers cures.

### 8.2 Experimental reproducibility

When discussing experimental reproducibility, special attention must be given to the vast number of variables that are modulated within experiments. As shown by the Sections above, there are endless attributes that can be modified independently and in conjunction with one another. Because of this, it is difficult to re-create a particular model used by another lab in order to directly verify a result. However, the variability between such models can be viewed as an advantage to the field. When two independent systems experimentally prod a specific characteristic of the tumor model and conclude the same or similar response tendency, i.e. reproducing each other’s findings, the result can be classified as robust. For example, serial 3D culture of ovarian cancer spheroids derived from cell lines as well as patient derived CSCs has been shown to increase resistance to cisplatin when formed via multiple different sphere formation methods including in 3D hanging drop plates[32], in low-attachment plates[293], and in traditional 2D culture with spontaneous spheroid budding[293] in separate labs. This conclusion being supported across multiple platforms is thus more reliable than a single platform alone. However, we find this is not always the case, and often similar experiments show contradictory results. This was the case when two separate labs knocked down ALDH1A1 with shRNA in ovarian cancer cell lines[294] or patient-derived ovarian cancer spheroids[295], showing that ALDH1A1 decreases[294] and increases[295] proliferation. This lack of reproducibility between labs draws attention to results which need to be investigated further. Much of this type of discrepancy can be attributed to unspecified conditions or slight differences in protocol that can have large effects on experimental outcome unbeknownst to the researchers.

To mitigate this conflict and improve overall reproducibility within the field, a more thorough and transparent documentation system should be practiced. Researchers should include detailed reports of their procedural characteristics and experimental failures as well. This would save not only time but also valuable funding resources and effort. Open source sharing of methodologies and recipes will help to establish consistency across experiments and facilitate best practices for studying a given microenvironment characteristic.

In peer-reviewed publications, reporting standards should be mandatory for experimental conditions, such as mechanical properties of the 3D culture system material (e.g. stiffness, porosity, permeability, and applicable rheometric values), biochemical properties of the model set up (e.g. cell adherence capabilities, oxygen diffusivity, and explicit medium compositions), and a thorough analysis of cellular profiles (e.g. genetic profile, cell subtype identification, plating densities, cell passage numbers, ratios of cell types). With this meticulous documentation, previously contradictory findings may be resolved and shed light on otherwise unidentified mechanisms within the TME. Additionally, the development of a standardized 3D model system that allows for fine control of each of the key TME characteristics (e.g. mechanical stimuli, co-culture, induced chemical gradients) would potentially eliminate dissimilar findings due to variation in experimental setups from different research groups.

### 8.3 Integration of biology and engineering

In the last 15 years, many transformative technologies have been developed to enhance the understanding of fundamental cancer biology and clinical translation. However, the integration of technologies and cancer biology has not been seamless, and as a result, the field has not made accelerated progress. Advances in molecular biology techniques allow for single cell downstream analysis; however, some techniques still require large number of cells like FACS, qRTPCR, and Western blot. Providing samples for such analysis without compromising the ability of an *in vitro* model to mimic the physiological TME is still technically challenging, cumbersome, and labor and time intensive. A key limitation to analysis techniques that require large cell numbers is the scarcity of patient samples, which most accurately represent *in vivo* phenotypes. To address the limitations imposed by assays that require large cell numbers to maintain accuracy, it is imperative to develop analysis methods that use low cell numbers. This will help to conserve rare cells like those available from patient samples and allow for multiple, high throughput, single cell assays, and effectively increase the information that can be obtained from a single patient sample. The effectiveness of this approach will improve with further development of minimalistic analysis techniques, such as drop sequencing, a single cell sequencing method, and automated liquid robotics enabled Western blot, which curtails sample volume needed for Western blotting.

There is also the issue of robustness of data acquired by different researchers for a single experiment, as user-to-user variability in technique leads to deviation in data. Success of the experiment depends on user experience, and most methods require learning new skills to ensure uniformity, robustness, and reproducibility. Often these skills become so specific to a subject that crossing the technological hurdles between biological and engineering fields becomes extremely difficult at a time when interdisciplinary research is essential for progress. This limitation highlights the need for detailed documentation of the methodologies discussed above. Often times, method sections will omit key experimental details that leave the reader extrapolating to fill in the gaps, which could lead to improper technique, unsuccessful experiments, and incongruent results, especially when interpreted by a less experienced researcher. As such, great care should be taken in drafting detailed methods that provide step-by-step instructions that researchers in different fields can follow. Such detailed methods will also help minimize experimental variation between researchers probing the same question. In addition, comprehensive reporting of model characterization will help to identify potential sources of variation between models from different research labs and thus aide in the accurate interpretation of results.

Furthermore, interdisciplinary efforts between dissimilar fields will help ensure a more seamless translation of model and analysis techniques between disciplines. For example, many physiologic models of cancers have been designed in engineering, physics, and chemistry labs with the intention of being widely adopted by the biology and clinical labs. However, the engineering parameters (such as fluid flow, ECM mechanics, etc.) might prove to be challenging to modulate for the biological and clinical labs. Evidence of this synergy between disciplines is already seen in collaborative grants, conferences, and publications that are being led by increasingly integrative teams in cancer biology, engineering, and oncology. One way to promote collaboration is through the promotion and development of more multidisciplinary conferences, which focus specifically on the inclusion of other fields to improve current models and techniques. These cross-disciplinary interactions are essential for the progress of the field and the improved design of experiments.

The power of interdisciplinary research in precision medicine is further evident in the development of advanced analytical techniques, which utilize concepts from physics, chemistry, and engineering to improve biological analysis of the more complex 3D *in vitro* tumor models present in research today. One such technique is live cell imaging performed with light sheet microscopy, which enables real-time live cell tracking and complete 3D imaging of the culture environment with minimal photobleaching, phototoxicity, and imaging time[296]. The information gleaned from such a technique may provide insight into key cell-cell interactions involved in tumor development and differentiation pathways and may help to explain experimental variation attributable to differences in 3D architecture[296], which would not otherwise be easily obtainable. Mass spectrometry-based proteomics is another advanced analytical technique that is becoming more widely implemented due to improvements in the mass spectrometry workflow and is now considered an irreplaceable molecular and cellular biology tool[297]. The powerful biological information provided by these advanced analysis techniques and other advanced imaging and ‘-omics’ analysis techniques in cancer research highlights the importance of interdisciplinary collaboration in enhancing progress in precision medicine[298].

Finally, increased development and utilization of mathematical models across subjects may serve as a common language through which biology, engineering, and chemistry can collaborate remotely, implement data to make predictions, and accelerate progress. The use of mathematical models in all fields is beneficial, as they can be created to analyze complex variables not easily studied experimentally, to determine which experiments are most promising, and to improve our understanding of biological mechanisms. These models could also decrease the use of scarce patient samples in experiments that can be determined *in situ* to not work and decrease the time needed to devise patient specific treatment plans. Integration will also be facilitated by the ease of sharing of codes used to generate mathematical models, effectively minimizing variance attributed to user differences and experience.

## Conclusion

Overall, cancer can be considered a complex and interconnected organ system that colludes with its host in order to progress and maintain function. Our understanding of the ‘cancer-organ’ system relies on our ability to produce experimental models that accurately replicate critical aspects of the TME and provide reliable and meaningful results. In order to complete this task, cancer bioengineering models should consider the three dimensionalities of the tumor, the mechanical stimuli that continuously provoke response, the multicellular interactions innate to the environment, and the variety of sources that can provide signaling to a heterogeneous tumor. Understandably, each of these aspects encompasses numerous degrees of freedom, complicating the overarching picture. To remedy these challenges, we propose: 1) Enhancing physiological reproducibility through development of more comprehensive *in vitro* models, 2) Improving experimental reproducibility via reporting standards and sharing of negative results, 3) Sharing of knowledge and expertise across fields through collaboration, and 4) Improvement of analysis techniques to reduce technological hurdles. These improvements will facilitate the use of integrated platforms for studying tumor progression and organization, and developing the next generation of ‘cancer-organ’ models. Moreover, as a result of this work, we will gain significant understanding regarding the complex ways in which cancer cells interact with their surroundings. This has direct implications for both effective cancer prevention and individualized therapies and achieving better patient survival.
